# Round Faces Are Associated with Sweet Foods: The Role of Crossmodal Correspondence in Social Perception

**DOI:** 10.3390/foods8030103

**Published:** 2019-03-19

**Authors:** Kosuke Motoki, Toshiki Saito, Rui Nouchi, Ryuta Kawashima, Motoaki Sugiura

**Affiliations:** 1 Institute of Development, Aging and Cancer, Tohoku University, Sendai 980-8575, Japan; toshiki.saito@med.tohoku.ac.jp (T.S.); rui.nouchi.a4@tohoku.ac.jp (R.N.); ryuta@tohoku.ac.jp (R.K.); sugiura@tohoku.ac.jp (M.S.); 2 Japan Society for the Promotion of Science, Tokyo 102-0083, Japan; 3 Smart-Aging Research Center, Tohoku University, Sendai 980-8575, Japan; 4 International Research Institute of Disaster Science, Tohoku University, Sendai 980-8575, Japan

**Keywords:** crossmodal correspondence, social judgment, facial shapes, sweet, sour

## Abstract

In retail settings, social perception of other peoples’ preferences is fundamental to successful interpersonal interactions (e.g., product recommendations, gift-giving). This type of perception must be made with little information, very often based solely on facial cues. Although people are capable of accurately predicting others’ preferences from facial cues, we do not yet know how such inferences are made by crossmodal correspondence (arbitrary sensory associations) between facial cues and inferred attributes. The crossmodal correspondence literature implies the existence of sensory associations between shapes and tastes, and people consistently match roundness and angularity to sweet and sour foods, respectively. Given that peoples’ faces have dimensions characterized by roundness and angularity, it may be plausible that people infer others’ preferences by relying on the correspondence between facial roundness and taste. Based on a crossmodal correspondence framework, this study aimed to reveal the role of shape–taste correspondences in social perception. We investigated whether Japanese participants infer others’ taste (sweet/sour) preferences based on facial shapes (roundness/angularity). The results showed that participants reliably inferred that round-faced (vs. angular-faced) individuals preferred sweet foods (Study 1). Round-faced individuals and sweet foods were well matched, and the matching mediated the inference of other person’s preferences (Study 2). An association between facial roundness and inference of sweet taste preferences was observed in more natural faces, and perceived obesity mediated this association (Study 3). These findings advance the applicability of crossmodal correspondences in social perception, and imply the pervasiveness of prejudicial bias in the marketplace.

## 1. Introduction

In retail settings, social perception of other peoples’ preferences is fundamental to successful interpersonal interactions. People often have opportunities to infer other peoples’ taste preferences during the process of product recommendation or gift-giving. Sales people may infer a consumer’s taste preferences when recommending food products. People give gifts of food to friends, family, or romantic partners by inferring the receiver’s taste on occasions such as anniversaries and annual events (birthdays, Christmas, or Valentine’s Day) [1]. In addition, inference of food preferences may have evolved from an adaptive advantage borne of food sharing (i.e., an instance of reciprocal altruism) and may later have extended to the domain of mate selection [2]. For example, we often infer taste preferences and prepare meals to share with others in a variety of situations (e.g., dating at restaurants, a dinner party with friends or guests). Together, inference of taste preferences forms a fundamental basis for retail and customer service settings. Although inference of others’ preferences is often erroneous and biased in the marketplace [3], we do not yet know how inferences regarding taste preferences are made. 

People make inferences about the attributes of individuals based on their faces. Although the face occupies a small area relative to the entire body, it plays a crucial role in social perception [4]. People are capable of determining attributes, such as gender, ethnicity, and age, from faces [4]. Additionally, people can accurately predict a variety of internal traits and characteristics, such as political attitudes [5], sexual orientation [6], the financial performance of CEOs [7], and social class [8], based on faces. As a result, faces provide a rich source of information about social attributes. However, we do not yet understand the complexities of how people infer preferences from faces. 

The implied relationships between faces and social attributes are based on sensory associations. Interactions between different senses are called crossmodal correspondences [9,10,11]. An example would be shape–sound correspondences. People reliably match certain sounds with certain visual characteristics, like roundness or sharpness. The arbitrary word “Bouba” tends to be assigned to round objects, whereas the word “Kiki” is likely to be associated with sharp objects [12]. This association may be linked to the way mouth movements generate the words; pronouncing the word “Bouba” requires rounding in the mouth, whereas saying “Kiki” involves a “sharp” movement. Based on shape–sound correspondences, a previous study showed that facial roundness is matched with social attributes, such as names that are pronounced by rounding the mouth (e.g., Bob—round face, Nick—angular face) [13]. Therefore, it is plausible that, like geometric shapes, people’s faces are likely to be matched with social attributes in line with crossmodal correspondence.

The term “social perception” can be defined as the initial stage of evaluating the intentions and psychological dispositions of others [14]. Social perception can be conceptualized as part of mentalizing [14], which is the perception of the dispositions and intentions of other individuals [15]. Thus, the domain of social perception is implicated when a study includes human features (e.g., faces) and/or social judgments (e.g., inference about a person’s preferences) [14,16,17]. However, it remains unknown whether correspondences between facial shapes and other senses (e.g., taste) influence inferences about other peoples’ preferences. This study investigated the role of facial shape and taste correspondence on social perceptions in the form of inferences about other peoples’ preferences.

Consistent with crossmodal correspondence, an inference about taste preference may be derived from facial roundness. The crossmodal correspondence literature suggests that people consistently match specific tastes to particular visual shapes [18]. Sweet food is reliably matched with roundness, while sour food is significantly fitted with angularity [19]. Thus, we hypothesized that people infer that round-faced (or angular-faced) people prefer sweet (or sour) foods. 

An inference of another’s preference is likely to be derived from shape–taste correspondences. Crossmodal correspondences are defined as the perceived matching of different senses [20], and shape–taste correspondences are also defined as matching of taste and shape [18]. In addition, there are individual differences in how people perceive the match between roundness/angularity and sweet/sour [18]. Thus, if an inference regarding another’s preference were based on a crossmodal correspondence framework, facial roundness and taste information should be matched, and the matching would mediate the inference of the other person’s preference. In other words, people who tend to perceive a match between round (or angular) and sweet (or sour) are more likely to infer that round- (or angular)-faced people prefer sweet (or sour) food. 

We investigated whether facial shape–taste correspondence influenced inferences about other people’s preferences. Study 1 examined whether individuals with round (vs. angular) faces would be inferred to have sweet (vs. sour) preferences. To show that the preference inference was based on shape–taste correspondences, Study 2 tested whether facial roundness and taste information were well matched, and whether that matching mediated the inference about the other person’s preferences. Using more realistic facial stimuli, Study 3 investigated which physical and/or personality characteristics related to facial roundness mediated associations between facial roundness and taste information. 

## 2. Study 1: Inference That Round-Faced People Prefer Sweet and Angular-Faced People Prefer Sour Tastes

Our first study examined whether individuals with round (vs. angular) faces would be assumed to prefer sweet (vs. sour) tastes.

### 2.1. Design

The study had a 2 (face: round, angular) × 2 (taste: sweet, sour) mixed-subject design, with face representing the between-subject and taste representing the within-subject factor. The main outcome was inferences about another person’s (taste) preference, which were made using a seven-point scale ranging from 1 = “Not at all” to 7 = “Very much”. 

### 2.2. Participants

In total, 56 healthy participants (27 females, *M*_age_ = 21.1 years old (*SD* = 2.0)) were recruited using a bulletin-board posting and a student mailing list email. This study was approved by the ethics committee of the School of Medicine at Tohoku University and was conducted in accordance with the Declaration of Helsinki. 

### 2.3. Task: Inference of Another Person’s Preference

Participants were randomly distributed into round (*n* = 27, 12 females) and angular face (*n* = 29, 15 females) groups. The round and angular faces were created using an online face generator (PimpTheFace), which was derived from a previous study that investigated correspondences between round/angular faces and sounds [13]. The face generator allows users to create round/angular facial stimuli with almost the same arrangement of inner parts (eyes, mouth, nose), and prevents possible confounding effects derived from facial parts, facial expressions, or skin color. 

**Figure 1:**
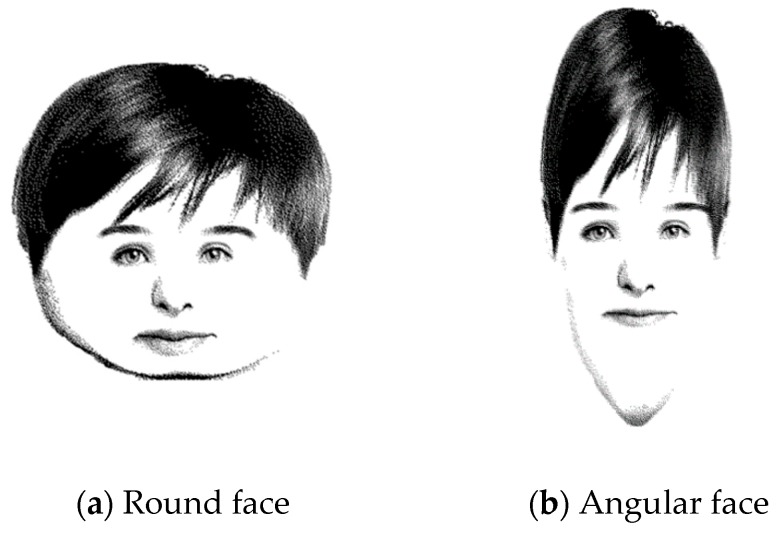
Round and angular faces presented to participants.

Groups of two to four participants were placed in a room that accommodated a maximum of 10 people. Across groups, participants were shown a drawing of a face and asked to consider the preferences of the person shown (Figure 1). Participants saw the face shown in Figure 1. To prevent possible influence of the person’s characteristics unrelated to roundness, the characteristics attributed to the person (male, age 20, 170 cm in height, 60 kg in weight, university student) were the same across groups except for facial roundness. The participants were given these attributes before answering the following questions. 

First, participants were asked to infer the preferences of each round- or angular-faced individual. The questions were: “How much do you think this person likes sweet foods?” and “How much do you think this person likes sour foods?”. Respondents provided their ratings for each question using a seven-point scale ranging from 1 = “Not at all” to 7 = “Very much”. 

Then, participants were asked to answer two questions: “How much do you like this person?” and “How round/angular is this person’s face?” The participants rated liking and roundness based on the conditions allocated to them (the round face or the angular face). The participants replied with their rating for the liking question using a seven-point scale from 1 = “Not at all” to 7 = “Very much” and for the round/angular question using a seven-point scale from 1 = “Very angular” to 7 = “Very round”. Responses to these questions were used to confirm the similarity of the preferences according to the facial shape and to replicate the finding regarding the differences in the perceived roundness of round and angular faces. A total of four questions were presented to participants. The questions were presented in a paper format and the order of questions was not randomized. It took participants a few minutes to complete the questions. 

### 2.4. Statistical Analysis

We applied analysis of variance (ANOVA) to assess the effects of facial shapes on inference of another person’s preference (sweetness, sourness). The design format was 2 (facial shape: round, angular) × 2 (tastes: sweet, sour), in which facial shapes functioned as the between-participants factor and taste as the within-participant factor. The inference of another person’s preference (“How much do you think this person likes sweet/sour foods?”), rated on a scale of 1–7, was the dependent variable used for ANOVA. The analyses were conducted using R ver. 3.3.1 and the R function “anovakun” ver. 4.8.0.

### 2.5. Results and Discussion

#### 2.5.1. Inference of Another Person’s Preference

There was no main effect of facial shape (*F*_1, 54_ = 0.007, *p* = 0.932, *η*^2^*p* = 0.0001). However, the main effect of taste was significant (*F*_1, 54_ = 66.444, *p* < 0.001, *η*^2^*p* = 0.552), indicating that participants believed that more people preferred sweet than preferred sour tastes. 

**Figure 2:**
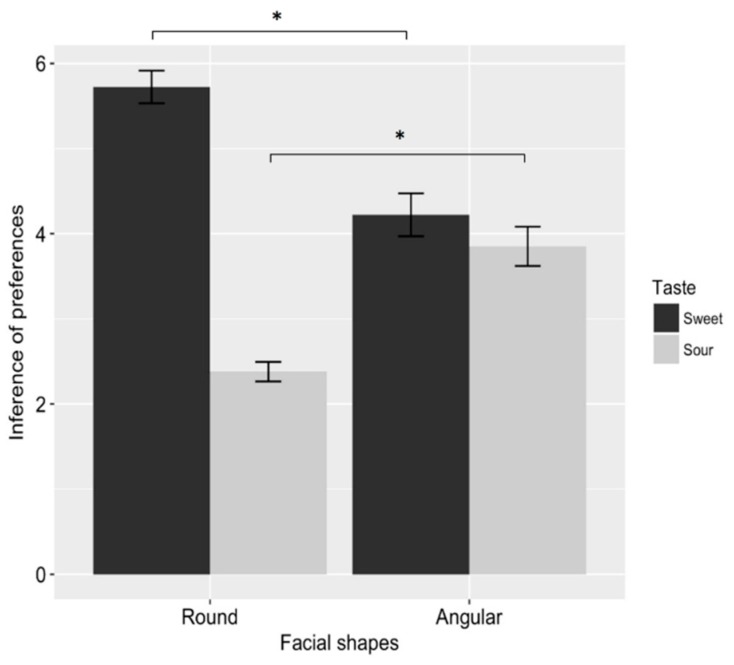
Results of Study 1. The round-faced person was perceived as more strongly preferring sweet (vs. sour) foods, while the angular-faced (vs. round-face) person was perceived as more strongly preferring sour (vs. sweet) foods. Error bars represent standard error. Asterisks denote significant difference (*p* < 0.05 *).

Notably, and consistent with our prediction, there was a significant interaction between face and taste (*F*_1, 54_ = 42.590, *p* < 0.001, *η*^2^*p* = 0.441). A planned comparison showed that participants inferred that round-faced (vs. angular-faced) individuals preferred sweet foods (round-faced_sweet_: *M* = 5.724, *SD* = 1.032 vs. angular-faced _sweet_: *M* = 4.222, *SD* =1.311, *F*_1, 54_ = 22.874, *p* < 0.001, *η*^2^*p* = 0.298), while angular-faced (vs. round-face) individuals preferred sour foods (angular-faced_sour_: *M* = 3.852, *SD* = 1.200 vs. round-faced_sour_: *M* = 2.379, *SD* = 0.622, *F*_1, 54_ = 33.947, *p* < 0.001, *η*^2^*p* = 0.386). The results showed that participants inferred that round- (vs. angular) faced individuals preferred sweet foods (vs. sour foods). The results are shown in Figure 2. 

#### 2.5.2. Liking and Perceive Roundness

Using a paired *t*-test, we compared preferences and perceived roundness between a round-faced person and an angular-faced person. There was no significant difference in preferences between a round-faced person and an angular-faced person (*M_round_* = 3.172 vs. *M_angular_* = 3.333; *t*_54_ = −0.419, *p* = 0.681). However, a round-faced person was perceived as having a rounder face than the angular-faced person (*M_round_* = 6.483 vs. *M_angular_* = 1.851; *t*_54_ = 16.907, *p* < 0.001). 

## 3. Study 2: Facial Roundness and Taste Information Matching, and Matching as a Mediator for Inference of Preferences

Our second study examined whether facial roundness and taste information were well matched and whether the match modulated the inference of another person’s preferences. The methods were similar to Study 1 except for the additional questions (“How much do you think this person is associated with sweet/sour foods?”). Respondents provided their ratings for each question using a seven-point scale ranging from 1 = “Not at all” to 7 = “Very much”.

### 3.1. Participants

In total, 41 healthy participants (18 females, *M*_age_ = 21.4 years old, *SD* = 1.9) were recruited using a bulletin-board posting and a student mailing list email. This study was approved by the ethics committee of the School of Medicine at Tohoku University and was conducted in accordance with the Declaration of Helsinki. Participants were randomly distributed into round- (*n* = 21, 8 females) and angular- (*n* = 20, 10 females) faced groups.

### 3.2. Statistical Analysis

To assess facial shape effects on inference of another person’s taste preferences, we applied ANOVA. The design format was 2 (facial shape: round, angular) × 2 (tastes: sweet, sour), in which facial shapes functioned as the between-participants factor and taste as the within-participant factor. The inference of another person’s preference was the dependent variable for ANOVA.

To assess facial shape effects on perceived matching with tastes, we applied ANOVA. The design format was 2 (facial shape: round, angular) × 2 (tastes: sweet, sour), in which facial shapes functioned as the between-participants factor and taste as the within-participant factors. We used perceived matching (“How much do you think this person is associated with sweet/sour foods?”), rated on a scale of 1–7, as the dependent variable for ANOVA.

To ascertain whether perceived matching mediated the relation between facial shape and inferences of sweet/sour preference, we conducted mediation analysis using the PROCESS macro for SPSS [21] and performed bootstrapping analyses using 5000 bootstrap samples [22]. Entering face shapes (roundness = 1, angularity = 0) as the independent variable (X), sweet preference as the outcome variable (Y), and perceived matching between shape and sweet food as the mediator variable (*M*), we estimated indirect effects using unstandardized regression coefficients. The Sobel test was used to evaluate the significance of indirect effects based on a normal theory approach [23].

### 3.3. Results and Discussion

#### 3.3.1. Inference of Other Peoples’ Preferences

There was no main effect of facial shape (*F*_1, 39_ = 1.797, *p* = 0.188, *η*^2^*p* = 0.044). However, the main effect of taste was significant (*F*_1, 39_ = 22.215, *p* < 0.001, *η*^2^*p* = 0.363), showing that participants inferred that more people preferred sweet tastes than sour ones.

**Figure 3:**
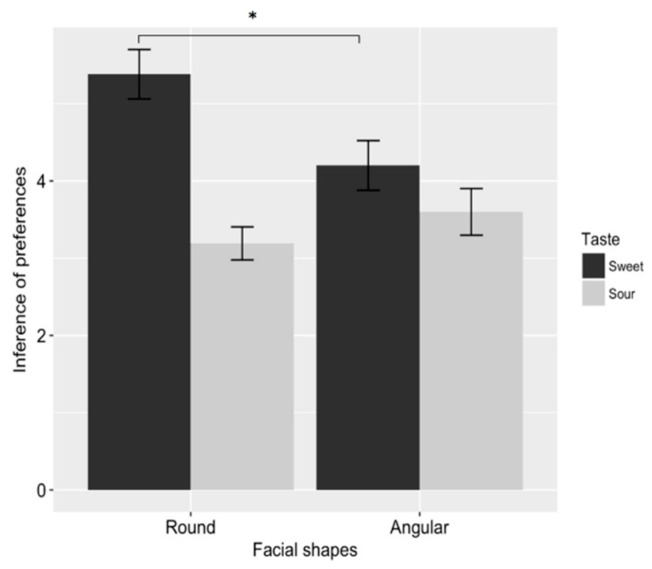
Results of Study 2. The round-faced (vs. angular-faced) person was perceived as more strongly preferring sweet (vs. sour) foods, while the angular-faced person was not to be perceived as liking sour (vs. sweet) foods. Error bars represent standard error. Asterisks denote significant difference (*p* < 0.05 *).

Results replicated those of Study 1, showing a significant interaction between face shape and taste (*F*_1, 39_ = 7.217, *p* = 0.011, *η*^2^*p* = 0.156). Planned comparisons showed that participants inferred that a round-faced (vs. angular-faced) person preferred sweet foods (round-faced_sweet_: *M* = 5.381, *SD* = 1.466 vs. angular-faced_sweet_: *M* = 4.200, *SD* = 1.436, *F*_1, 39_ = 6.782, *p* = 0.013, *η*^2^*p* = 0.148), while an angular-faced (vs. round-faced) person preferred sour foods (angular-faced_sour_: *M* = 3.600, *SD* = 1.353 vs. round-faced_sour_: *M* = 3.190, *SD* = 0.981, *F*_1, 39_ = 1.240, *p* = 0.272, *η*^2^*p* = 0.031). The results are shown in Figure 3. 

#### 3.3.2. Matching Facial Roundness to Taste Information

There was no main effect of facial shape, *F*_1, 39_ = 1.976, *p* = 0.168, *η*^2^*p* = 0.048. However, the main effect of taste was significant (*F*_1, 39_ = 19.409, *p* < 0.001, *η*^2^*p* = 0.332). Such that participants perceived that people generally prefer sweet tastes over sour ones.

There was a significant interaction between face shape and taste (*F*_1, 39_ = 10.939, *p* = 0.002, *η*^2^*p* = 0.219). A planned comparison showed that a round-faced (vs. angular-faced) person was more frequently matched with sweet foods (round-faced _sweet_: *M* = 5.191, *SD* = 1.537 vs. angular-faced_sweet_: *M* = 3.600, *SD* =1.667, *F*_1, 39_ = 10.102, *p* = 0.003, *η*^2^*p* = 0.206), while the match between an angular-faced person (vs. round-faced person) and sour foods was marginally significant (angular-faced_sour_: *M* = 3.200, *SD* =1.642 vs. round-faced_sour_: *M* = 2.380, *SD* = 0.865, *F*_1, 39_ = 4.051, *p* = 0.051, *η*^2^*p* = 0.094).

#### 3.3.3. Perceived Matching as a Mediator

To examine whether perceived matching mediated the relationship between facial shape and inferences of sweet/sour preference, we conducted a mediational analysis. We modeled the indirect effect of facial shape on inferences about round-faced people’s preferences as mediated by perceived matching.

**Figure 4:**
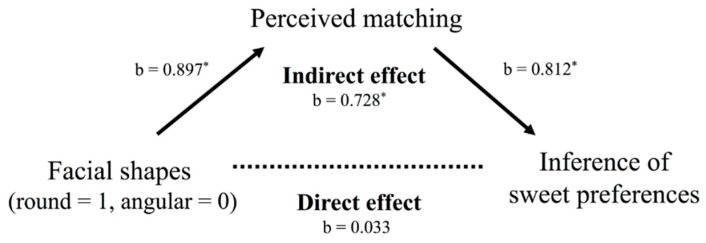
Model to predict the effect of facial shapes on inference of sweet preference mediated by perceived matching. Standardized coefficients (b) are displayed. Asterisks indicate significant paths (*p* < 0.05 *).

Supporting the prediction, the bootstrap estimates were positive, and the 95% bias-corrected confidence intervals did not include zero. The total indirect effect was 0.761, *SE* = 0.206, *CI* (0.302, 1.266). The significance of the indirect effect was confirmed by the Sobel test (*z* = 2.992, *p* = 0.004) (Figure 4).

#### 3.3.4. Preference and Perceived Roundness

Using a paired *t*-test, we compared the preferences and perceived roundness of round-faced and angular-faced persons. There was no significant difference in preferences between the round-faced and angular-faced persons (*M_round_* = 3.810 vs. *M_angular_* = 3.300; *t*_39_ = 1.065, *p* = 0.293). However, the round-faced person was perceived as having a rounder face than the angular-faced person (*M_round_* = 6.476 vs. *M_angular_* = 1.950; *t*_39_ = 12.770, *p* < 0.001).

## 4. Study 3: Facial Roundness and Taste Information Matching Based on More Realistic Faces, and the Physical and/or Personality Characteristics Related to Facial Roundness as Mediators for Inference of Preferences

In a third study, we aimed to (1) detect the mediator of facial roundness and inference of other’s sweet preference and (2) increase the reliability of our findings. Although Study 2 showed that perceived matching mediated sensory associations, the mechanism underlying the perceived matching remains elusive. One possible explanation why people form impressions based on facial roundness–taste correspondences may pertain to the physical and/or personality characteristics related to facial roundness (obesity, happiness, extraversion) (e.g., [24]). People may expect round-faced people to be more obese, happy, likable, and extraverted, leading to inferences about preferences biased toward sweet foods. To investigate this possibility, we asked participants to rate the perceived obesity, happiness, likability, and extraversion of each face. Furthermore, to ensure that our findings in Studies 1–2 were not stimuli-specific (two cartoon faces with extremely round and extremely angular faces were used), we tried to replicate our findings using a variety of more realistic facial stimuli (24 faces with moderately round, neutral, and moderately angular faces). Moreover, we added the other taste preferences (saltiness, bitterness) to confirm the specificity of the associations between facial roundness and inferences about taste preferences.

### 4.1. Design

The study had a 3 (face: round, angular, neutral) × 4 (taste: sweet, sour, salty, bitter) within-subject design, treating both face and taste as within-subject factors. The main outcome was the inference of (taste) preference. 

### 4.2. Participants

In total, 33 healthy participants (17 females, *M*_age_ = 36.8 years old, *SD* = 9.2) were recruited through Lancers and completed the survey on Qualtrics. The sample size was determined using G*power software [25]. We entered the medium effect size (f = 0.25), alpha level (a = 0.05/4, because we have four measures, sweet, sour, salty, and bitter, in Study 3), and power (1-b = 0.80) into G*Power. The required sample size was 30; we used a sample size of 33. The populations differed from those in Study 1 and 2, to increase the generalizability of our findings. This study was approved by the Ethics Committee of the School of Medicine at Tohoku University and was conducted in accordance with the Declaration of Helsinki. 

### 4.3. Task: Inference of Another Person’s Preference

**Figure 5:**
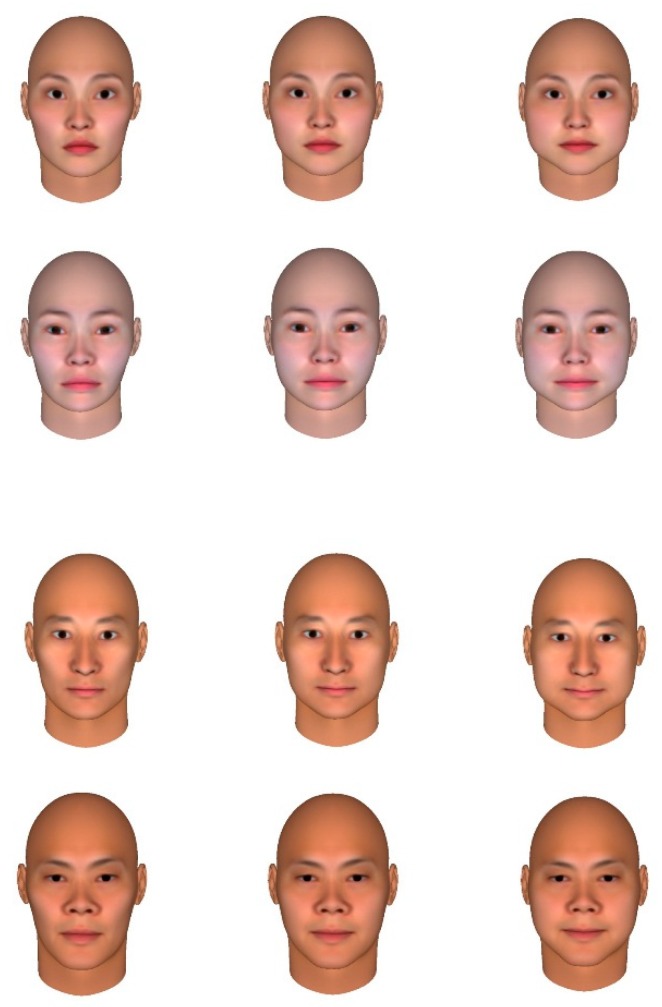
Examples of angular faces, neutral faces, and round faces are on the left, middle, and right, respectively.

Following previous studies [26,27] (FaceGen Modeller 3.14), we created the round, angular, and neutral faces using a realistic 3D human face generator. Initially, we randomly generated four male and four female faces (Asian) for the neutral conditions. Using FaceGen Modeller, we changed the cheeks (round/gaunt) parameter for the round faces (the parameter = −0.5), neutral faces (the parameter = 0), and angular faces (the parameter = +0.5). FaceGen Modeller allows users to create round/angular facial stimuli with an almost identical arrangement of inner features (eyes, mouth, nose) and prevents possible confounding effects derived from facial parts, facial expressions, or skin color. We used 24 stimuli (four males and four females ranging from round-faced to neutral to angular-faced). Examples of stimuli used in Study 3 are shown in Figure 5. 

First, participants were asked to infer the taste preferences (sweet, sour, salty, and bitter) of the facial images: “How much do you think this person likes sweet foods?”, “How much do you think this person likes sour foods?”, “How much do you think this person likes salty foods?”, and “How much do you think this person likes bitter foods?” Respondents provided their ratings for each question using a visual analogue scale (VAS) ranging from 0 = “Not at all” to 100 = “Very much”. The order of questions was randomized.

Next, participants were asked to rate the obesity, happiness, extraversion, and likability of the individuals: “How obese is this person?”, “How happy is this person?”, “How extraverted is this person?”, and “How much do you like this person?” using a VAS scale ranging from 0 = “not at all” to 100 = “very much”. The order of the four questions was randomized. Finally, participants were asked to rate the roundness/angularity of each face (“How round/angular is this person’s face?”) on a VAS scale ranging from 0 = “very angular” to 100 = “very round”.

### 4.4. Statistical Analysis

To assess facial shape effects on inference of another person’s taste preferences, we applied ANOVA. The design format was 3 (facial shape: round, angular, neutral) × 2 (tastes: sweet, sour, salty, bitter), in which facial shapes and taste as the within-participant factor. Inference of another person’s preferences was the dependent variable for ANOVA. For cases in which a significant interaction was found, we conducted post-hoc analyses to evaluate the interaction. Post-hoc analyses were conducted using Shaffer’s modified sequentially rejective Bonferroni test procedure. The analyses were conducted using R ver. 3.3.1 and the R function “anovakun” ver. 4.8.0.

To ascertain details of the effects of facial shapes on the inference of another person’s taste preferences, we conducted regression analyses. First, to test the relations between facial roundness and taste, we performed single regression analyses using inferences about preferences for respective tastes (sweet, sour, salty, bitter) as predictors and perceived roundness (rated on a VAS from very angular = 0 to very round = 100) as explanatory variables. The analysis was restricted to those tastes for which significance was found using ANOVA analysis.

Second, to test the relations between the inference of another person’s taste preferences and physical or personality characteristics related to facial roundness (obesity, happiness, extraversion, and likability), we conducted single regression analyses using inferences about preferences for respective tastes as predictors and physical or personality characteristics related to facial roundness (obesity, happiness, extraversion, and likability) as explanatory variables.

Next, we ran multiple regression analysis to search for variables influencing inferences about taste preferences by including perceived roundness and physical or personality characteristics related to facial roundness in the model. We used inferences about taste preferences as predictors, perceived roundness as an explanatory variable, and physical or personality characteristics related to facial roundness (obesity, happiness, extraversion, and likability) as control variables. Regression analyses were conducted using R ver. 3.3.1 and the R function “lmres”.

To assess whether physical or personality characteristics related to facial roundness (obesity, happiness, extraversion, and likability) mediate the relation between facial shape and inferences of taste preferences, we conducted our mediation analysis using the PROCESS macro for SPSS [21] with 5000 bootstrap samples [22]. Entering perceived roundness as the independent variable (X), inferences of taste preferences as the outcome variable (Y), and the physical or personality characteristics related to facial roundness (obesity, happiness, extraversion, and likability) as the mediator variable (*M*) or control variables, we estimated indirect effects using unstandardized regression coefficients. The Sobel test was applied to assess the significance of indirect effects based on a normal theory approach [23]. All tests were two-tailed. Statistical significance was inferred for *p* < 0.05.

### 4.5. Results and Discussion

#### 4.5.1. Inferences about Preferences

There was no main effect of facial shape (*F*_2, 64_ = 1.801, *p* = 0.173, *η*^2^*p* = 0.053). However, the main effect of taste was significant (*F*_3, 96_ = 17.637, *p* < 0.001, *η*^2^*p* = 0.355), indicating that participants inferred that people prefer certain tastes over others. Pairwise analyses showed that people inferred that sweet foods (*M* = 54.117, *SD* = 12.132) were more strongly preferred than bitter foods (*M* = 46.934, *SD* = 11.058, *t*_32_ = 3.594, *adj.p* = 0.003). There were no differences between sweet foods and sour foods (*M* = 52.230, *SD* = 11.102, *t*_32_ = 1.116, *adj.p* = 0.273). People inferred that salty foods (*M* = 62.408, *SD* = 11.071) were more strongly preferred than sweet foods (*t*_32_ = 3.884, *adj.p* = 0.002), sour foods (*t*_32_ = 4.072, *adj.p* < 0.001), and bitter foods (*t*_32_ = 5.627, *adj.p* < 0.001). People inferred that sour foods were more strongly preferred than bitter foods (*t*_32_ = 3.141, *adj.p* = 0.007). 

**Figure 6:**
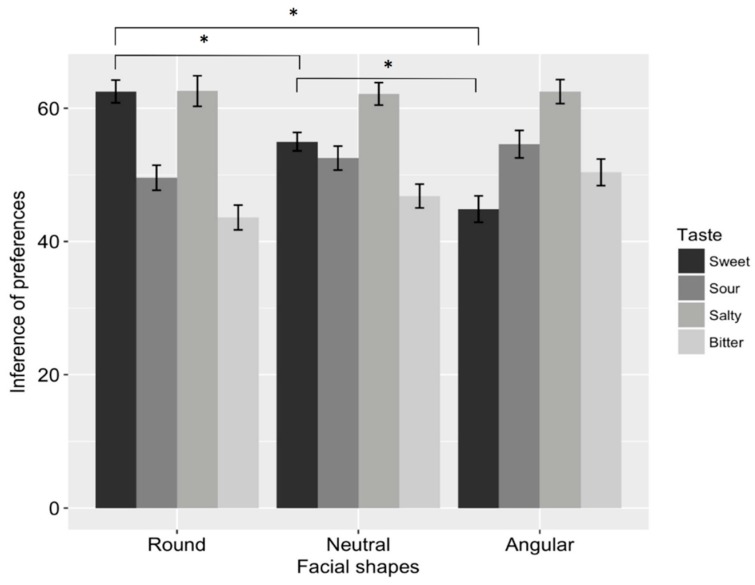
Results of Study 3. Round-faced (vs. angular- and neutral-faced) individuals were perceived as more strongly preferring sweet foods. Angular-faced (vs. round- and neutral-faced) individuals were perceived as more strongly preferring bitter foods. Angular-faced (vs. round-faced) individuals were perceived as more strongly preferring sour foods. Asterisks indicate significant paths (*p* < 0.05 *). Error bars represent standard error.

Importantly, these results replicated those of Studies 1–2 and reflected a significant interaction between face shape and taste (*F*_6, 192_ = 29.993, *p* < 0.001, *η*^2^*p* = 0.484). Post hoc analyses showed the simple effects of the interaction between face shape and taste (Figure 6). Different facial shapes differentially influenced inferences about preferences for sweet (*F*_2, 64_ = 44.372, *p* < 0.001, *η*^2^*p* = 0.581), sour (*F*_2, 64_ = 9.018, *p* < 0.001, *η*^2^*p* = 0.220), and bitter (*F*_2, 64_ = 11.772, *p* < 0.001, *η*^2^*p* = 0.269) foods, but it did not have a significant effect on preferences for salty foods (*F*_2, 64_ = 0.053, *p* = 0.949, *η*^2^*p* = 0.002). 

Planned comparisons showed that participants inferred that round-faced people (*M* = 62.511, *SD* = 9.801) preferred sweet foods more strongly than did angular-faced (*M* = 54.981, *SD* = 7.955, *t*_32_ = 7.215, *adj.p* < 0.001) and neutral-faced (*M* = 44.860, *SD* = 11.384, *t*_32_ = 6.236, *adj.p* < 0.001) people. They also inferred that neutral-faced people preferred sweet foods more strongly than did angular-faced people (*t*_32_ = 5.339, *adj.p* < 0.001). The results are shown in Figure 6.

Participants inferred that angular-faced people (*M* = 54.599, *SD* = 11.874) preferred sour foods more strongly than round-faced people did (*M* = 49.572, *SD* = 10.782, *t*_32_ = 3.950, *adj.p* = 0.001). They also inferred that neutral-faced people (*M* = 52.512, *SD* = 10.349) preferred sour foods more strongly than did round-faced people (*t*_32_ = 2.732, *adj.p* = 0.010). The differences between angular- and neutral-faced people were not significant (*t*_32_ = 1.720, *adj.p* < 0.095). 

Participants inferred that angular-faced people (*M* = 50.379, *SD* = 11.457) preferred bitter foods more strongly than did round-faced (*M* = 43.599, *SD* = 10.696, *t*_32_ = 4.920, *adj.p* = 0.001) and neutral-faced (*M* = 46.826, *SD* = 10.252, *t*_32_ = 2.508, *adj.p* = 0.017) people. They also inferred that neutral-faced people preferred bitter foods more strongly than did round-faced people (*t*_32_ = 2.308, *adj.p* = 0.028).

#### 4.5.2. Predicting Inferences about Taste Preference from Facial Roundness

**Figure 7:**
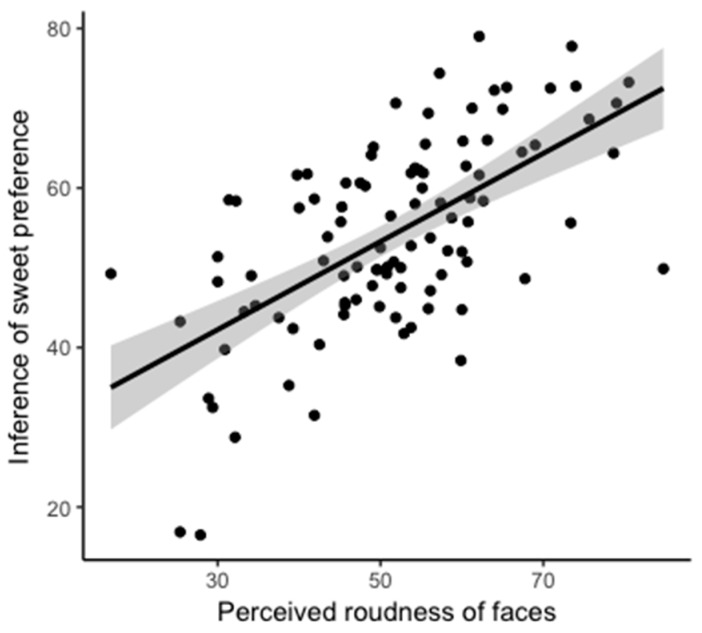
Scatterplots show correlations between the perceived roundness of faces and inferences about preferences for sweet foods. The dots were derived from the mean value of each condition (round, angular, and neutral) for each participant. This graph is for illustration purposes only, as we did not control for potential confounding factors (*t* = 7.719, *p* < 0.001, r_97_ = 0.617).

Perceived facial roundness significantly explained inferences about sweet (β = 0.552, *SE* = 0.0716, *t* = 7.719, *p* < 0.001; Figure 7) and salty (β = −0.239, *SE* = 0.079, *t* = −3.012, *p* = 0.003) preferences but not sour (β = −0.155, *SE* =0.082, *t* = −1.900, *p* = 0.060) or bitter (β = 0.087, *SE* = 0.083, *t* = 1.049, *p* = 0.297) preferences.

#### 4.5.3. Predicting Inferences about Taste Preference from Physical and/or Personality Characteristics Related to Facial Roundness

**Figure A1:**
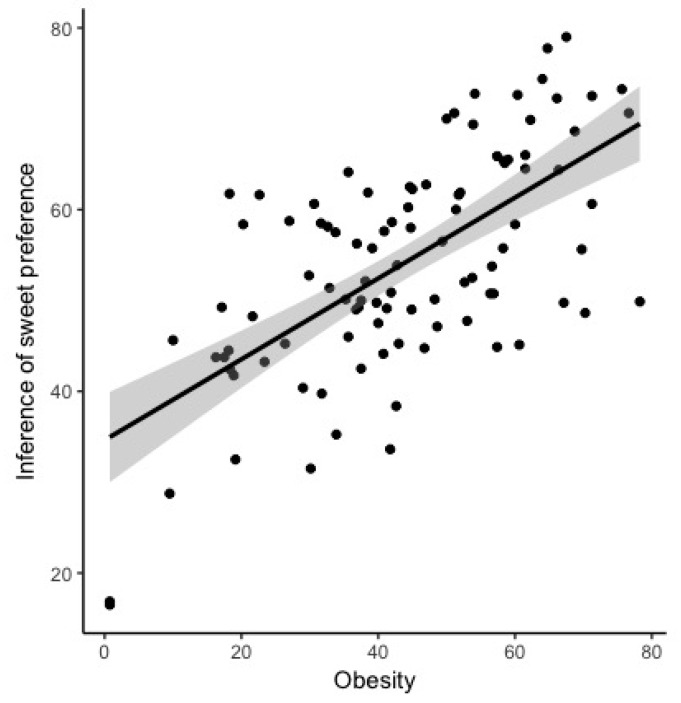
Scatterplot of the relationship between perceived obesity and inferences of sweet preference.

**Figure A2:**
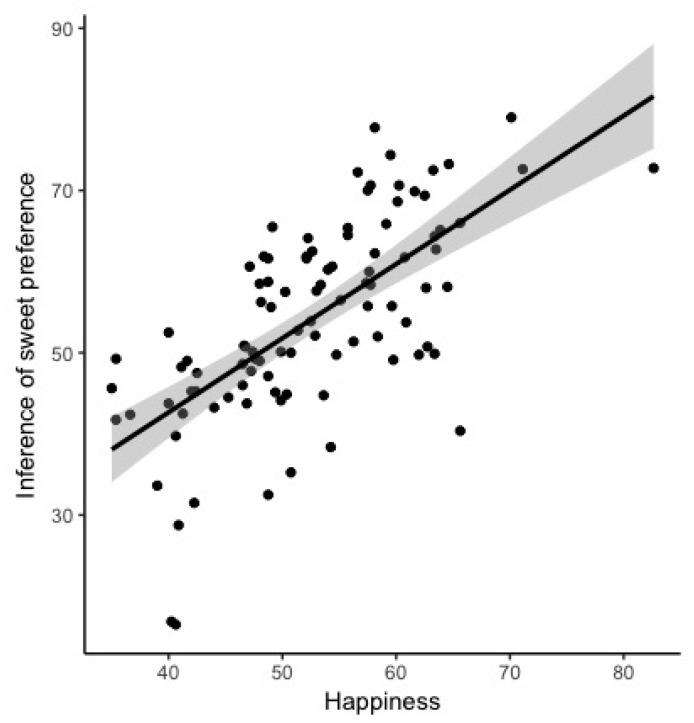
Scatterplot of the relationship between perceived happiness and inferences of sweet preference.

**Figure A3:**
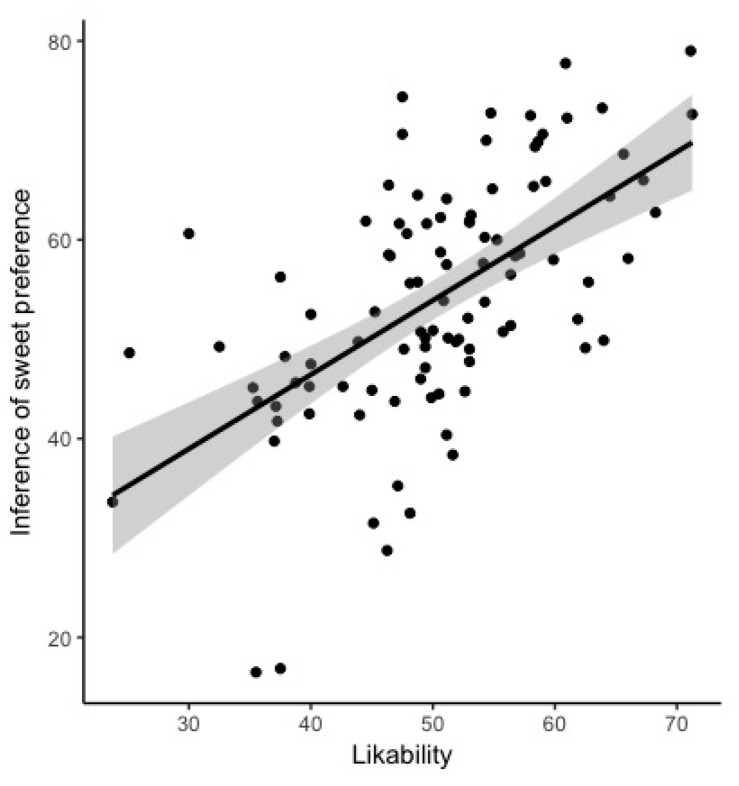
Scatterplot of the relationship between likability and inferences of sweet preference.

**Figure A4:**
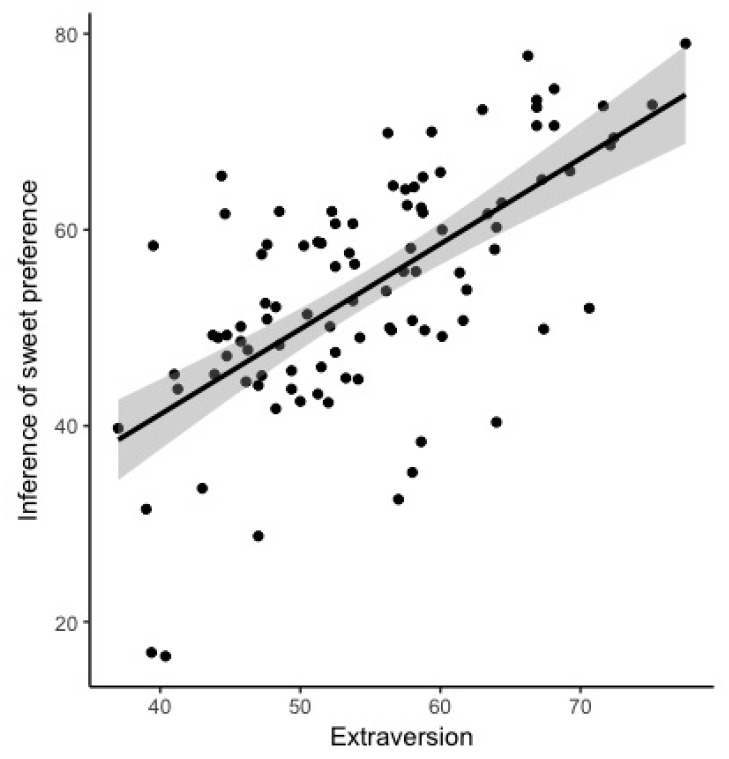
Scatterplot of the relationship between perceived extraversion and inferences of sweet preference.

All of the variables (obesity, happiness, extraversion, and likability) significantly explained inferences about sweetness preference (obesity: β = 0.642, *SE* = 0.078, *t* = 8.254, *p* < 0.001; happiness: β = 0.666, *SE* = 0.076, *t* = 8.799, *p* < 0.001; extraversion: β = 0.649, *SE* = 0.077, *t* = 8.394, *p* < 0.001; likability: β = 0.583, *SE* = 0.083, *t* = 7.061, *p* = 0.297). The scatterplots are shown in Appendix A Figure A1, Figure A2, Figure A3 and Figure A4. 

#### 4.5.4. Predicting Inferences about Sweet Preference from Facial Roundness after Controlling for Physical and/or Personality Characteristics Related to Facial Roundness

After controlling physical and/or personality characteristics related to facial roundness (obesity, happiness, extraversion, and likability), perceived roundness did not independently contribute to inferences about sweet preferences (β = 0.262, *SE* = 0.1102, *t* = 2.378, *p* = 0.019). Happiness, extraversion, and likability did not contribute to inferences about sweet preferences (happiness: β = 0.262, *SE* = 0.1102, *t* = 2.378, *p* = 0.019; extraversion: β = 0.262, *SE* = 0.1102, *t* = 2.378, *p* = 0.019; likability: β = 0.262, *SE* = 0.1102, *t* = 2.378, *p* = 0.019). Only obesity was significantly related to inferences about sweet preferences (β = 0.262, *SE* = 0.1102, *t* = 2.378, *p* = 0.019). 

#### 4.5.5. Obesity as a Mediator

To examine whether obesity mediates the relationship between facial shape and inferences of sweet/sour preferences, we carried out a mediational analysis. We modeled the indirect effect of facial shape on inferences about round-faced people’s sweet preferences, as mediated by obesity after controlling for happiness, extraversion, and likability. 

**Figure 8:**
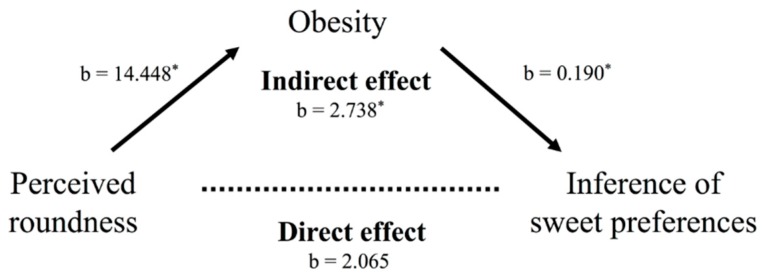
Model to predict the effect of facial shape on inference of sweet preference mediated by perceived matching. Standardized coefficients (b) are displayed. Asterisks indicate significant paths (*p* < 0.05 *).

**Figure A5:**
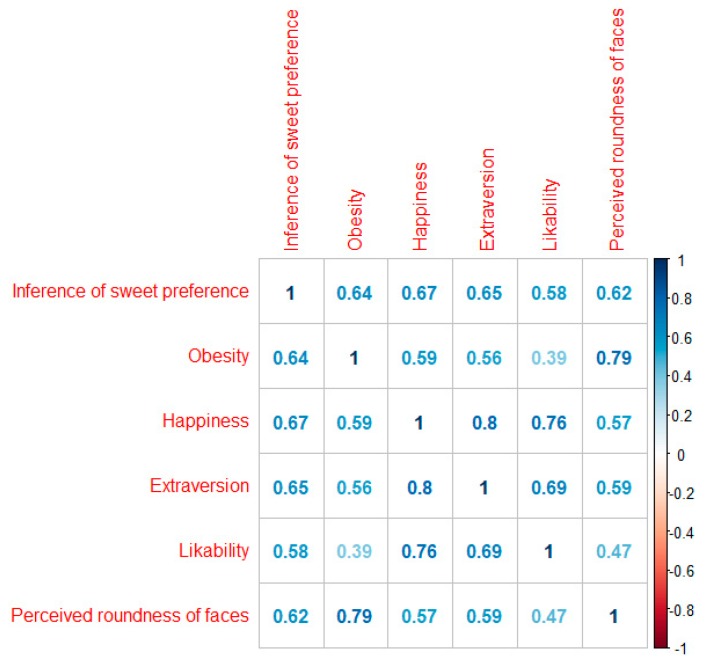
The correlation matrix; all measures showed positive correlations.

Supporting the prediction, the bootstrap estimates were positive, and the 95% bias-corrected confidence intervals did not include zero. The total indirect effect was 2.738, *SE* = 1.234, *CI* (0.103, 5.488). The significance of the indirect effect was confirmed by the Sobel test (*z* = 2.201, *p* = 0.023) (Figure 8). A correlation matrix of all variables is shown in Appendix A Figure A5.

## 5. Discussion

### 5.1. Overview of the Findings

In this study, we conducted three experiments designed to investigate the role of taste–shape correspondence in social perception. Relying on a crossmodal correspondence framework, we investigated whether inferences about other peoples’ preferences are made using sensory associations between facial roundness and sweet taste. The results of Study 1 demonstrated that people infer that round-faced individuals are more likely to prefer sweet food. The results of Study 2 showed that rounder faces and sweet foods were well matched, indicating that facial roundness and sweet taste corresponded. In addition, this matching mediated the inference of another person’s preferences. People who experienced a greater match between facial shapes and tastes were more likely to infer that round-faced individuals preferred sweet foods. The results of Study 3 replicated associations between facial roundness and sweet taste using more natural faces, and found that perceived obesity mediated the associations. It seems that people inferred that round-faced (vs. neutral-faced or angular-faced) individuals preferred sweet foods using physical clues related to obesity. These findings indicate that people evaluate social attributes based on facial shape in line with crossmodal correspondences.

### 5.2. Theoretical Contributions

The results showed that arbitrary matching between facial features and attributes influenced impression formation. It has been proposed that facial features can influence impression formation through overgeneralization effects. For example, attractiveness can be overgeneralized so that people with more attractive faces are perceived as having attractive (positive) qualities on a host of dimensions [17]. However, our findings cannot be explained by overgeneralization effects. It is unlikely that facial roundness can be generalized to inferences of sweet preference (since sweet is not literally “round” taste). Alternatively, relying on a crossmodal correspondence framework, our findings showed that a good match (but not an overgeneralization) between facial roundness (visual shape) and sweetness (taste) influences impression formation. Taken together, consistent with the literature on shape-taste correspondences [3,28], the findings indicate that multi-modal matching of facial roundness (visual shape) with sweetness (taste) has an impact on impression formation.

Why is facial roundness related to an inference of sweet preferences? It has been suggested that three mechanisms may underlay this crossmodal correspondence: structural, statistical, and semantic [11]. It seems that the statistical explanation supports our findings. People may experience and/or learn that round-faced individuals prefer sweet foods. For example, babies, who have rounder faces, prefer sweet tastes (and show positive facial expressions when exposed to sweet tastes) [29]. Although the individuals with round and angular faces were the same age in our study, the participants might explicitly or implicitly associate rounder-faced individuals with babies who respond favorably to sweetness. Furthermore, obese people, who are also characterized by facial roundness, prefer sweet foods [30]. People often observe that obese people prefer sweets, in real life or in the media. Participants may make base inferences about other people’s preferences based on their experience. Study 3 supported the possibility that characteristics of obese people, such as facial roundness, inform inferences about other people’s sweet preferences. 

Although facial roundness and sweet correspondence influenced social perception, facial sharpness and sour correspondence did not. Study 1 showed that participants inferred that individuals with angular faces preferred sour food, but this finding was not replicated in Study 2. Moreover, the match between angular-faced individuals and sour food was not significant (there was only a slight trend). Study 3 also found that, after controlling for confounding factors, participants did not infer that individuals with angular faces preferred sour food. This may be because humans generally do not like sour food. People do not often give sour food as gifts, and we have less opportunity to infer other peoples’ sour preferences. Thus, participants showed less matching between faces and sour tastes regardless of their roundness or angularity. 

This study was limited by not controlling for the degree of human-likeness between round-faced and angular-faced individuals. Although the degree of human-likeness of the faces was not controlled, scores of liking for round and angular faces were determined. Human-likeness and liking measures are highly correlated (e.g., rs 0.5–0.6 discussed in a study on the degree of human-likeness of faces [31]). In this study, the degree of liking did not differ between the round face and the angular face (Study 1: M_round_ = 3.172 vs. M_angular_ = 3.333; *t_54_* = −0.419, *p* = 0.681; and Study 2: M_round_ = 3.810 vs. M_angular_ = 3.300; *t_39_* = 1.065, *p* = 0.293). Although the preference for round and angular faces significantly differed, Study 3 found that participants inferred that individuals with round faces preferred sweet food when we controlled for liking. Thus, the results are not likely to be influenced by the differences in human-likeness. However, it remains an open question as to whether these findings were affected by the human-likeness of the faces presented. Further study is needed to determine whether results can be replicated when controlling for human-likeness. 

### 5.3. Issues Relevant to Consumer Behaviors

The findings have implications for practitioners. Food store managers can train sales people not to recommend products based on facial shape–obesity–sweet associations. At restaurants, customers sometimes ask waiters “What do you recommend?” A waiter might respond with “I recommend this food” based on the customer’s physical attributes. Physical stereotypes in marketplace discrimination, which involves stereotyped treatment of customers [32], is common in encounters with the service industry. Employees therein sometimes base their recommendations on stereotypes [3]. For example, a recent study showed that sales agents are more likely to recommend round (vs. angular) products to obese consumers [3]. Given our findings, sales agents may recommend sweet foods to round-faced consumers according to their perceptions of obesity. If a round-faced person was made aware that he/she received product recommendations based on facial shape–obesity–sweet associations, that person might feel uncomfortable. Awareness of prejudicial treatment in the marketplace reduces consumer purchasing and the intention to revisit a retailer [33]. To avoid negative consequences, food store managers should incorporate issues of stereotypes based on facial shape–obesity–sweet associations in their sales training.

### 5.4. Limitations and Future Directions

There were limitations to this study. Firstly, the participants were all Japanese, and the findings might therefore be culture-specific. A range of cross-cultural differences have been documented for sensory associations [34,35,36]. For example, Western participants associated carbonated/still water with angular/round shapes, while participants from rural Namibia did not infer any such associations [34]. However, cross-cultural similarities have also been reported for taste-shape associations [34,36]. British and Colombian participants associated sweet juice with round shapes [19]. Rounder typefaces were rated as sweeter by participants from Colombia, the United Kingdom, and China [35]. Together, the data suggest that rounder shapes are reliably associated with sweet tastes across cultures, and rounder facial shapes may also be reliably mapped on to sweet tastes, regardless of culture. However, there is no direct evidence regarding cross-cultural differences and similarities in facial shape-taste associations. Further research is needed to investigate how people from different cultures perceive associations between facial shape and sweet taste. Secondly, the possibility exists of other mediators influencing relations between perceived facial roundness and perceived preferences for sweet tastes. We used obesity, extraversion, happiness, and likability as potential mediators, and identified obesity as mediating relations between perceived roundness of the face and perceived preference for sweet tastes after controlling for other factors. However, other variables (e.g., babyish features) not included in this study might mediate relations between perceived roundness and sweet preferences inferred for others. Further research must be conducted to detect other potential mediators. Thirdly, our sample sizes are small. Given the recent replication crisis, further research is necessary to replicate our findings using a larger sample size.

Our findings open the door for future empirical tests and the role of other crossmodal correspondences in inferences regarding other peoples’ preferences. Shape can be matched with sound, and this shape–sound correspondence may influence inferences of other peoples’ preferences. For example, roundness has been shown to be associated with higher pitches [37]. Thus, people may infer that round-faced people prefer high-pitched music. In addition, it has been shown that round-shaped faces are associated with names whose pronunciation requires rounding of the mouth (e.g., “Bouba”) [12]. Thus, people may infer that round-faced individuals prefer brand names that require a rounding of the mouth to pronounce. These research questions regarding the role of other crossmodal correspondences in inferences regarding other peoples’ preferences should be investigated.

## References

[1] Gino F., Flynn F.J. (2011). Give them what they want: The benefits of explicitness in gift exchange. J. Exp. Soc. Psychol..

[2] Saad G., Gill T. (2003). An evolutionary psychology perspective on gift giving among young adults. Psychol. Mark..

[3] Vallen B., Sridhar K., Rubin D., Ilyuk V., Block L.G., Argo J.J. (2018). Shape-and Trait-Congruency: Using Appearance-based Cues as a Basis for Product Recommendations. J. Consum. Psychol..

[4] Todorov A., Olivola C.Y., Dotsch R., Mende-Siedlecki P. (2015). Social attributions from faces: Determinants, consequences, accuracy, and functional significance. Annu. Rev. Psychol..

[5] Rule N.O., Ambady N. (2010). Democrats and Republicans can be differentiated from their faces. PLoS ONE.

[6] Rule N.O., Ambady N., Hallett K.C. (2009). Female sexual orientation is perceived accurately, rapidly, and automatically from the face and its features. J. Expe. Soc. Psychol..

[7] Wong E.M., Ormiston M.E., Haselhuhn M.P. (2011). A face only an investor could love: CEOs’ facial structure predicts their firms’ financial performance. Psychol. Sci..

[8] Bjornsdottir R.T., Rule N.O. (2017). The Visibility of Social Class From Facial Cues. J. Personal. Soc. Psychol..

[9] Motoki K., Saito T., Nouchi R., Kawashima R., Sugiura M. A Sweet Voice: The Influence of Crossmodal Correspondences Between Taste and Vocal Pitch on Advertising Effectiveness. https://www.researchgate.net/publication/329466113_A_sweet_voice_The_influence_of_crossmodal_correspondences_between_taste_and_vocal_pitch_on_advertising_effectiveness.

[10] Motoki K., Saito T., Nouchi R., Kawashima R., Sugiura M. (2019). Light colors and comfortable warmth: Crossmodal correspondences between thermal sensations and color lightness influence consumer behavior. Food Qual. Prefer..

[11] Spence C. (2011). Crossmodal correspondences: A tutorial review. Atten Percept Psychophys.

[12] Ramachandran V.S., Hubbard E.M. (2001). Synaesthesia-a window into perception, thought and language. J. Consciou. Stud..

[13] Barton D.N., Halberstadt J. (2017). A social Bouba/Kiki effect: A bias for people whose names match their faces. Psychon. Bull. Rev..

[14] Allison T., Puce A., McCarthy G. (2000). Social perception from visual cues: role of the STS region. Trend. Cognit. Sci..

[15] Frith C.D., Frith U. (1999). Interacting minds-a biological basis. Science.

[16] Ames D.L., Fiske S.T., Todorov A.T. (2011). 28 Impression Formation: A Focus on Others’ Intents. The Oxford Handbook of Social Neuroscience.

[17] Zebrowitz L.A., Montepare J.M. (2008). Social psychological face perception: Why appearance matters. Soc. Pers. Psychol..

[18] Velasco C., Woods A.T., Petit O., Cheok A.D., Spence C. (2016). Crossmodal correspondences between taste and shape, and their implications for product packaging: a review. Food Qual. Prefer..

[19] Ngo M.K., Velasco C., Salgado A., Boehm E., O’Neill D., Spence C. (2013). Assessing crossmodal correspondences in exotic fruit juices: The case of shape and sound symbolism. Food Qual. Prefer..

[20] Spence C. (2012). Managing sensory expectations concerning products and brands: Capitalizing on the potential of sound and shape symbolism. J. Consum. Psychol..

[21] Hayes A.F. (2013). Introduction to Mediation, Moderation, and Conditional Process Analysis: A Regression-Based Approach.

[22] Preacher K.J., Hayes A.F. (2008). Asymptotic and resampling strategies for assessing and comparing indirect effects in multiple mediator models. Behav. Res..

[23] Sobel M.E. (1982). Asymptotic confidence intervals for indirect effects in structural equation models. Sociol. Methodol..

[24] Okamura Y., Ura M. (2018). Shapes of faces and eyeglasses influence the judgement of facial impressions in a metaphor-consistent manner. Curr. Psychol..

[25] Faul F., Erdfelder E., Buchner A., Lang A.-G. (2009). Statistical power analyses using G* Power 3.1: Tests for correlation and regression analyses. Behav. Res..

[26] Oosterhof N.N., Todorov A. (2008). The functional basis of face evaluation. Proc. Natl. Acad. Sci..

[27] Saito T., Nouchi R., Kinjo H., Kawashima R. (2017). Gaze Bias in Preference Judgments by Younger and Older Adults. Front. Neuroinform..

[28] Velasco C., Woods A.T., DeRoy O., Spence C. (2015). Hedonic mediation of the crossmodal correspondence between taste and shape. Food Qual. Prefer..

[29] Steiner J. (1974). Discussion paper: innate, discriminative human facial expressions to taste and smell stimulation. Ann. N. Y. Acad. Sci..

[30] Frijters J.E.R., Rasmussen-Conrad E.L. (1982). Sensory discrimination, intensity perception, and affective judgment of sucrose-sweetness in the overweight. J. Gen. Psychol..

[31] Bruneau E., Jacoby N., Kteily N., Saxe R. (2018). Denying humanity: The distinct neural correlates of blatant dehumanization. J. Exp. Psychol. Gen..

[32] Walsh G. (2009). Disadvantaged consumers’ experiences of marketplace discrimination in customer services. J. Mark. Manag..

[33] King E.B., Shapiro J.R., Hebl M.R., Singletary S.L., Turner S. (2006). The stigma of obesity in customer service: A mechanism for remediation and bottom-line consequences of interpersonal discrimination. J. Appl. Psychol..

[34] Bremner A.J., Caparos S., Davidoff J., de Fockert J., Linnell K.J., Spence C. (2013). “Bouba” and “Kiki” in Namibia? A remote culture make similar shape–sound matches, but different shape–taste matches to Westerners. Cognition.

[35] Velasco C., Woods A.T., Wan X., Salgado-Montejo A., Bernal-Torres C., Cheok A.D., Spence C. (2017). The taste of typefaces in different countries and languages. Psychol. Aesthet. Creat. Arts.

[36] Wan X., Woods A.T., Bosch J.J.F.V.D., McKenzie K.J., Velasco C., Spence C. (2014). Cross-cultural differences in crossmodal correspondences between basic tastes and visual features. Front. Psychol..

[37] Wang Q.J., Wang S., Spence C. (2016). “Turn up the taste”: assessing the role of t-aste intensity and emotion in mediating crossmodal correspondences between basic tastes and pitch. Chem. Senses.

